# Characterization of Neutralizing Monoclonal Antibodies and Identification of a Novel Conserved C-Terminal Linear Epitope on the Hemagglutinin Protein of the H9N2 Avian Influenza Virus

**DOI:** 10.3390/v14112530

**Published:** 2022-11-15

**Authors:** Yanan Wang, Xueyang Li, Qianru Xu, Xiangxiang Niu, Shenli Zhang, Xiaotian Qu, Hongyan Chu, Jinxuan Chen, Qianqian Shi, Erqin Zhang, Gaiping Zhang

**Affiliations:** 1 College of Veterinary Medicine, Jilin University, Changchun 130062, China; 2 International Associated Research Center of National Animal Immunology, College of Veterinary Medicine, Henan Agricultural University, Zhengzhou 450046, China; 3 School of Basic Medical Sciences, Henan University, Kaifeng 475004, China; 4 Longhu Modern Immunity Laboratory, Zhengzhou 450046, China; 5 School of Advanced Agricultural Sciences, Peking University, Beijing 100871, China; 6 Henan Provincial Key Laboratory of Animal Immunology, Henan Academy of Agricultural Sciences, Zhengzhou 450002, China; 7 Jiangsu Co-Innovation Center for the Prevention and Control of Important Animal Infectious Disease and Zoonoses, Yangzhou University, Yangzhou 225000, China

**Keywords:** H9N2 avian influenza virus, hemagglutinin protein, neutralizing monoclonal antibodies, linear B cell epitope, HA2

## Abstract

The H9N2 avian influenza virus (AIV) remains a serious threat to the global poultry industry and public health. The hemagglutinin (HA) protein is an essential protective antigen of AIVs and a major target of neutralizing antibodies and vaccines. Therefore, in this study, we used rice-derived HA protein as an immunogen to generate monoclonal antibodies (mAbs) and screened them using an immunoperoxidase monolayer assay and indirect enzyme-linked immunosorbent assay. Eight mAbs reacted well with the recombinant H9N2 AIV and HA protein, four of which exhibited potent inhibitory activity against hemagglutination, while three showed remarkable neutralization capacities. Western blotting confirmed that two mAbs bound to the HA protein. Linear epitopes were identified using the mAbs; a novel linear epitope, ^480^HKCDDQCM^487^, was identified. Structural analysis revealed that the novel linear epitope is located at the C-terminus of HA2 near the disulfide bond-linked HA1 and HA2. Alignment of the amino acid sequences showed that the epitope was highly conserved among multiple H9N2 AIV strains. The results of this study provide novel insights for refining vaccine and diagnostic strategies and expand our understanding of the immune response against AIV.

## 1. Introduction

The H9N2 avian influenza virus (AIV) is a low-pathogenicity type A avian influenza virus and belongs to the Orthomyxoviridae family [1]. H9N2 AIV has been circulating globally and leads to severe disease and high mortality due to mixed infections with other pathogens [2,3], thus causing significant economic losses [4]. H9N2 has attracted substantial attention due to its wide host range, avian-to-human transmission, and genetic diversity owing to frequent antigenic drift or antigenic shift [5,6,7]. Evidence has shown that H9N2 donates internal genes to generate novel human-infecting AIVs, such as H7N9, H10N8, and H5N6 [8,9,10], thereby contributing to the emergence and evolution of reassortment AIVs, posing a persistent threat to public health [11].

Hemagglutinin (HA), the major surface glycoprotein of influenza viruses, is primarily involved in receptor binding and membrane fusion during influenza virus infection of host cells. HA is highly immunogenic and is considered the primary target for immune protection [12]. Prior to becoming a functionally active homologous trimeric molecule, HA exists in the form of a precursor named HA0, which undergoes proteolytic cleavage into HA1 and HA2 linked with a disulfide bond, thereby forming the globular head region and the stem region, respectively. The HA head includes a receptor-binding domain (RBD) that binds the virus to the sialic acid receptors of its target cells, while the HA stem region is primarily responsible for virus–host membrane fusion [13]. Several antibodies can bind to the HA globular head and prevent viral binding to host cells, such as the HA1-specific neutralizing monoclonal antibodies (mAbs) 5J8, C05, 1B2, and F005-126 [14,15,16,17]. However, the globular head region is variable, whereas the stem region is correspondingly conserved [18]. For example, the HA2-specific neutralizing mAbs CR626 and F10 recognize multiple AIV subtypes [19,20], while FI6v3, which elicits its neutralizing activity by binding to the F subdomain of the HA trimer, recognizes a conserved epitope of the H1-H16 influenza A subtypes [21]. Moreover, CR9114 binds a conserved epitope in the HA stem and protects both influenza A and B viruses [22].

These epitopes play important roles in AIV infection and provide candidates for developing universal influenza virus vaccines, establishing immunodetection technologies, and understanding the mechanisms of immune response to AIV. Unlike conformational epitopes, linear epitopes are composed of a continuous amino acid sequence of an antigen and function influenced by its primary structure. Changes in linear epitopes may cause structural and affiliative alternation in an antibody. Moreover, recent research on the B cell linear epitope landscape of the SARS-CoV-2 spike protein suggested correlation between linear epitope responses and disease severity and outcome [23]. However, to our knowledge, most available epitopes of the H9N2 HA protein are located at the HA1 region and fusion peptide and are rarely conserved among H9N2 subtype AIV. 

Therefore, in the present study, mice were immunized with recombinant HA protein expressed in rice endosperm to generate mAbs. These mAbs exhibited high reactivities with both H9N2 AIV and HA protein, with three mAbs displaying strong neutralization capacities. The generated mAbs recognized a novel B cell linear epitope of the H9N2 HA protein. The newly identified linear epitope ^480^HKCDDQCM^487^ is located at the C-terminal globule domain of the HA2 stem region, which may play an important role in membrane fusion. ^480^HKCDDQCM^487^ is a highly conserved epitope and an ideal candidate for the differential diagnosis of H9N2 AIV. Developing monoclonal antibodies and identifying B cell epitopes will provide a basis for diagnostic assays and therapeutic and prophylactic vaccine strategies against the H9N2 AIV. These findings will contribute to the prevention and control of influenza A virus infection.

## 2. Materials and Methods

### 2.1. Cells, Proteins, and Reagents

Madin–Daby canine kidney cells (MDCK) and SP2/0 mouse myeloma cells were provided by the Key Laboratory of Animal Immunology, Henan Academy of Agricultural Science. RPMI Medium 1640 and Dulbecco’s modified Eagle’s medium (DMEM) were obtained from Solarbio Life Sciences, China. Fetal bovine serum (FBS) was obtained from Moregate Biotech. Horseradish peroxidase (HRP)-conjugated goat anti-mice secondary antibodies were purchased from Jackson Immuno Research Laboratories, West Grove, PA, USA. All other chemicals used were of reagent grade. Recombinant HA protein expressed in the rice endosperm was provided by the International Associated Research Center of National Animal Immunology. 

### 2.2. Mouse Immunization and Generation of Monoclonal Antibodies

Female BALB/c mice (6–8 weeks old) were obtained from the Laboratory Animal Center of Zhengzhou University. The mice were immunized thrice subcutaneously with 100 μL purified HA protein (10 μg) mixed with equivalent Montanide ISA 50 V2 adjuvant (SEPPIC, Paris, France) at an interval of 2 weeks. The soluble protein extracted from TP309 (rice without expressing HA protein) was used as the negative control. Blood samples were collected and the serum was separated to determine the antibody titer via enzyme-linked immunosorbent assay (ELISA). Two weeks following the final immunization, booster immunization was conducted via intraperitoneal injection of 20 μg recombinant HA protein into mice with the highest antibody titers, and cell fusion was performed 3 days later as described previously [24]. Briefly, the spleen cells of the boosted immunized mouse were collected and fused with SP2/0 cells (at a ratio of 1:6) using PEG 1500. Subsequently, the fused cells were selected in hypoxanthine–aminopterin–thymidine (HAT) medium and hypoxanthine-thymidine (HT) medium. After 10 days, the supernatant was screened by indirect ELISA and immunoperoxidase monolayer assay (IPMA), and the positive cells were subcloned three times; the selected hybridoma cells were injected into the enterocoelia of BALB/c mice to obtain the ascitic fluid and the ascitic fluid were purified by protein G column chromatography (GE Healthcare, Waukesha, WI, USA) as the instructions. Isotypes of mAbs were determined using a mouse monoclonal antibody isotyping kit (Thermo Scientific, Waltham, MA, USA). 

### 2.3. IPMA Test 

The IPMA was used to screen for monoclonal antibodies. Briefly, MDCK cells were cultured in 96-well plates and infected with 100 μL H9N2 AIV at a concentration of 100 TCID_50_/0.1 mL. After incubation with FBS-free DMEM at 37 °C for 1 h in 5% CO_2_, the FBS-free medium was replaced with DMEM containing 1% FBS and incubated for another 18–20 h. All virus dilutions contained 2 μg/mL TPCK-trypsin. The plates were washed three times with PBST and fixed with 4% paraformaldehyde at −20 °C for 30 min. After washing three times with PBST, the cells were blocked with 5% skimmed milk in PBST at 37 °C for 1 h. Following washing with PBST, the plates were incubated with hybridoma cells supernatant for 1 h at 37 °C. The plates were then washed three times with PBST and incubated with HRP-conjugated goat anti-mouse secondary antibody (1:5000) for 1 h at 37 °C. The AEC reagent was used for staining, and the cells were observed under a light microscope; the titer was the highest dilution of mAbs with observing the stained cells.

To compare the reactivity of the eight mAbs with the virus, an IPMA was conducted using purified ascitic fluid (0.1 mg/mL) and the purified ascitic fluid was serially double-diluted.

### 2.4. Indirect ELISA

Indirect ELISA was used to screen for mAbs. Briefly, 96-well plates were coated with purified HA protein (1 μg/mL) diluted with carbonate buffer at 4 °C overnight. After washing with PBST thrice, the plates were blocked with 5% skimmed milk in PBST at 37 ℃ for 2 h. The hybridoma cells supernatant, H9N2 AIV-positive serum (positive control), and negative serum were serially double-diluted from 1:400, added to the reaction plates, and incubated at 37 °C for 1 h. Next, the plates were washed five times with PBST and incubated with HRP-conjugated goat anti-mouse secondary antibody (1:5000) for 1 h at 37 °C. Finally, a TMB solution (Solarbio) was used as the chromogenic reagent and the reaction was stopped with 2 M H_2_SO_4_. Absorbance was measured at 450 nm using a microplate reader (Omega, Berlin, Germany). It was determined as positive when the hybridoma cells supernatant reached OD450nm/NC OD450 nm ≥ 2.1 (P/N ≥ 2.1); the titer was the highest dilution that can be judged as positive [25].

In order to compare the reaction of the eight mAbs with the HA protein, an indirect ELISA was performed using purified ascitic fluid (0.1 mg/mL) and the purified ascitic fluid was serially double-diluted.

To determine the binding affinity of mAbs, the Ka of mAbs was calculated according to the method proposed by Beatty et al. in 1987 [26]. The calculation formula is as follows:

Ka = (n − 1)/2(n[Ab’]t − [Ab]t)

n = [Ag]t/[Ag’]t

where [Ag]t and [Ag’]t stand for two different concentrations of coating antigen, respectively, HA protein with 0.5 μg/mL and 1 μg/mL in this study; [Ab]t and [Ab’]t express two different concentrations of the mAbs at 50% of the ODmax.

### 2.5. Microneutralization Assay

A microneutralization assay was performed to detect the neutralizing activity of the screened mAbs as described previously [27]. The serially double-diluted mAbs (initial concentration of 0.1 mg/mL) mixed with H9N2 AIV were incubated for 1 h at 37 ℃, and then added to MDCK cells and cultured for an additional 1 h. Subsequently, the FBS-free DMEM was replaced with DMEM containing 1% FBS, and the cells were incubated for 18–20 h. All virus dilutions contained 2 μg/mL TPCK-trypsin. After fixation and blocking, cells were incubated with the corresponding primary antibodies (1:10,000) and HRP-conjugated secondary antibodies (1:5000). Finally, the cells were stained with an AEC solution and examined under a light microscope. The titer was the highest dilution of mAbs without the stained cells.

### 2.6. Hemagglutination Inhibition Assay

A hemagglutination inhibition (HI) assay was conducted to detect the hemagglutination inhibitory activities of the generated mAbs as described previously [27]. The ascites fluid (initial concentration of 0.1 mg/mL) collected from the mice (50 μL) was serially double-diluted and added to microtiter plates. Next, 50 μL of the H9N2 AIV virus with 4 hemagglutination units (HAU) was added to the plates. The mixture was then vortexed and incubated for 40 min at room temperature, after which a 50 μL standardized solution of 1% red blood cells was added. Following incubation at room temperature for 30 min, HI titers were defined as the highest ascites fluid dilution that inhibited hemagglutination. Both positive and negative controls were included.

### 2.7. Western Blotting

Western blotting was conducted to analyze the reaction between the mAbs and purified HA protein under denaturing conditions as previously described [28]. HA protein and soluble protein extracted from TP309 (negative control) were separated using 10% SDS polyacrylamide gels and transferred to polyvinylidene difluoride (PVDF) membranes. The PVDF membranes were then blocked with 5% skim milk in PBST at 4 °C overnight. Next, the membranes were incubated with the mAbs (1:10,000) for 1 h at room temperature. The membranes were then washed with PBST three times and incubated with HRP-conjugated goat anti-mouse secondary antibody (1:5000) for 1 h at 37 ℃. Finally, the membranes were visualized using enhanced chemiluminescence (ECL) reagent (NCM Biotech, Shanghai, China) and viewed using a Fusion FX system (VILBER LOURMAT).

### 2.8. Dot Blotting

Dot blotting was performed to detect the specificity of the mAbs [29]. The inactivated H9N2 AIV, H5N1 AIV, NDV (Newcastle disease virus) and TP309 were spotted on nitrocellulose (NC) membranes (Millipore, Burlington, MA, USA). After drying at room temperature, the NC membranes were blocked overnight with 5% skim milk in PBST at 4 °C. The membranes were then incubated with the mAbs (1:10,000) for 1 h at room temperature, washed with PBST thrice, and incubated with HRP-conjugated goat anti-mouse secondary antibody (1:5000) for 1 h at room temperature. Finally, the membranes were visualized using an ECL reagent (NCM Biotech) with a Fusion FX system (VILBER LOURMAT).

### 2.9. Mapping the Linear B Cell Epitope of the Recombinant HA Protein

To map the linear epitopes, 41 overlapping peptides 20–23 amino acids long (containing an offset of 7 aa) were synthesized by GL Biochem (Shanghai, China; Table 1) according to the HA protein sequence (residues 19–521) derived from A/chicken/Henan/43/02 (H9N2) (GenBank Accession number AAY52507.1). The minimal peptides were synthesized by GL Biochem (Table 2). A cysteine (C) residue was added to each polypeptide to couple the peptides with bovine serum albumin (BSA). The purity of the peptides was > 95%. Dot blotting and indirect ELISA were performed to screen for synthetic peptides.

Dot blotting was performed as described previously. The soluble synthetic peptide (1 μg), purified HA protein (1 μg, positive control), and BSA (1 μg, negative control) were spotted on nitrocellulose (NC) membranes (Millipore, Burlington, MA, USA). The mAb 6H2 was used as the primary antibody (1:10,000).

Indirect ELISA was conducted as described above. Synthesized peptides (1 μg/mL) were used as coating antigens. The mAb 6H2 was used as primary antibody (1:10,000).

### 2.10. Structural Locations of Positive Peptides

The hemagglutinin structure (PDB: 1JSD) of the influenza A virus (A/swine/Hong Kong/9/98(H9N2)) was used as the template to locate the positive peptides. The spatial distribution of the novel epitope was determined via mapping the epitope on HA using the PyMOL Molecular Graphics System (Version 2.3.0, Schrödinger, LLC.).

### 2.11. Conservation Analysis

Epitope conservation analysis of the HA protein from different strains of H9N2 and H1-H18 subtypes was conducted via multiple sequence alignment using MEGA-X software (Institute of Molecular Evolutionary Genetics, The Pennsylvania State University, State College, PA, USA) and Jalview (Version:2.11.2.2; The Barton Group, University of Dundee, Dundee, UK).

## 3. Results

### 3.1. H9N2 HA Immunization and Generation of mAbs

**Figure 1:**
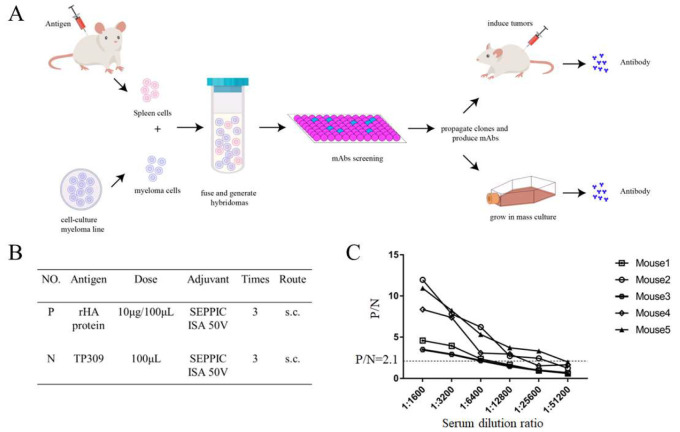
H9N2 HA immunization and process of generate mAbs. (**A**) Schedule of generate mAbs by hybridoma technology. BALB/c mice were immunized with HA antigen and generate mAbs. The spleen cells were fused with the myeloma cells and were screened, and finally hybridoma cells were propagated to produce mAbs. (**B**) Details of immunizing dose, adjuvant, frequency and routes. S.C represents subcutaneous injection. (**C**) HA-specific antibody of serum detected by indirect ELISA. N represents OD_450nm_ of mice sera immunized with TP309. P stands for OD_450nm_ of mice sera immunized with recombinant HA protein; P/N ≥ 2.1 is considered as positive. All the data are repeated three times and the average values are calculated.

Monoclonal antibodies were generated as illustrated in Figure 1A. BALB/c mice were immunized three times with purified HA protein (Figure 1B), following which blood samples were collected and the serum was separated. Figure 1C shows the serum (collected 1 week after last immunization) antibody titer detected via indirect ELISA, with the highest being 1:25,600. Spleen cells of the mouse with the highest serum antibody titer were prepared to fuse with SP2/0 mouse myeloma cells and generate hybridoma cells.

### 3.2. mAbs Screening

**Figure 2:**
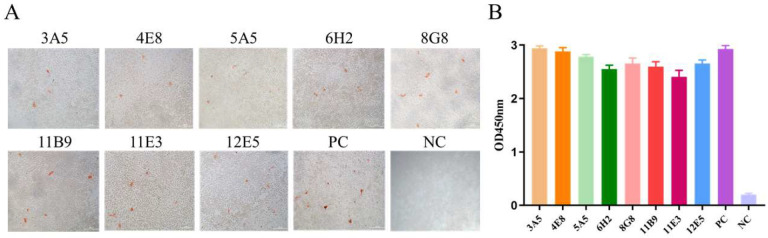
Screening of mAbs against the H9N2 AIV and HA protein. (**A**) Screening mAbs by IPMA. Eight mAbs, 3A5, 4E8, 5A5, 6H2, 8G8, 11B9, 11E3, and 12E5 were identified. (**B**) ELISA results for reaction of mAbs with the HA protein. PC, H9N2 AIV-positive chicken serum. NC, SP2/0 cell culture supernatants.

The IPMA test was conducted to screen the antibodies generated against H9N2 AIV. Eight monoclonal antibodies reacted with A/chicken/Henan/43/02 (H9N2) AIV strain-infected MDCK cells and were referred to as 3A5, 4E8, 5A5, 6H2, 8G8, 11B9, 11E3, and 12E5. The signals of H9N2 AIV and mAbs are shown in Figure 2A. The mAbs were serially double-diluted from 1:400 to the detected IPMA titer; the results showed the eight mAbs reacted strongly with the H9N2 AIV, of which mAbs 8G8 and 12E5 reacted until being diluted to 1:102,400 (Table 3). The results of the indirect ELISA (Figure 2B) suggest the specific and strong reactions between the mAbs and the HA protein; the highest antibody titer reached 1:204,800 (Table 3). To determine the binding affinity of mAbs, the Ka of mAbs was calculated and the results are shown in Table 3. Among these mAbs, the immunoglobulin isotypes of six mAbs were IgG1, except for 5A5, which was an IgG2a, and 11B9, which was an IgG2b; all light chains were Kappa (Table 3).

### 3.3. Characterization of the Generated mAbs

**Figure 3:**
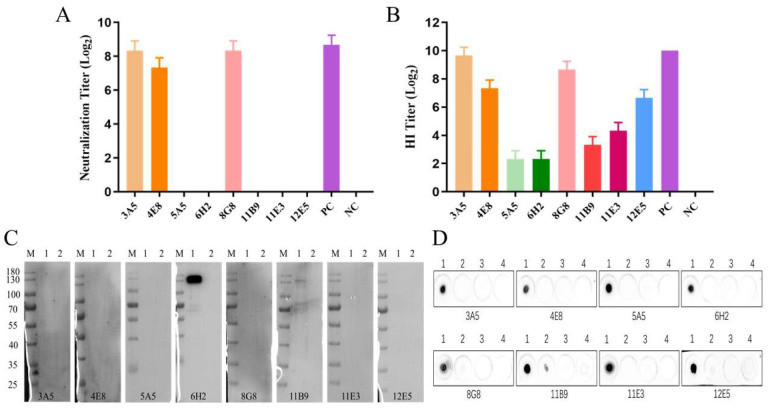
Identification of mAbs. (**A**) Neutralizing activity of mAbs. PC, H9N2 AIV-positive chicken serum. NC, H9N2 AIV-negative mice serum. (**B**) Hemagglutination inhibitory activity of mAbs. PC, H9N2 AIV-positive chicken serum. NC, H9N2 AIV-negative mice serum. (**C**) Evaluation of the response between the mAbs and HA protein by Western blotting. M, protein marker. Lane 1, HA protein. Lane 2, TP309 soluble protein used as negative control. (**D**) Specificity detection of the mAbs react with different virus by dot blotting. Lane 1, H9N2 AIV virus. Lane 2, H5N1 AIV virus. Lane 3, NDV virus. Line 4, TP309 soluble protein. All the data were presented as means with error bars.

We conducted a microneutralization assay and found that mAbs 3A5, 4E8, and 8G8 possessed remarkable neutralization capacities, with virus neutralization titers reaching 1:315 (Figure 3A). mAbs 3A5, 4E8, 8G8, and 12E5 showed strong inhibitory activity against hemagglutination, while the remaining four showed weak activity (Figure 3B). The results of Western blotting are presented in Figure 3C; both 6H2 and 11B9 reacted with the HA protein under denaturing conditions, indicating that these two mAbs recognized linear epitopes of the H9N2 HA protein. The results of dot blotting confirmed that the mAbs only reacted with H9N2 AIV, but not H5N1 AIV, NDV, and TP309; 11B9 had a weak reaction with H5N1 AIV and 8G8 and 12E5 also showed little weak binding to the H5N1 AIV (Figure 3D).

### 3.4. Mapping of B Cell Epitope on the HA Protein

**Figure 4:**
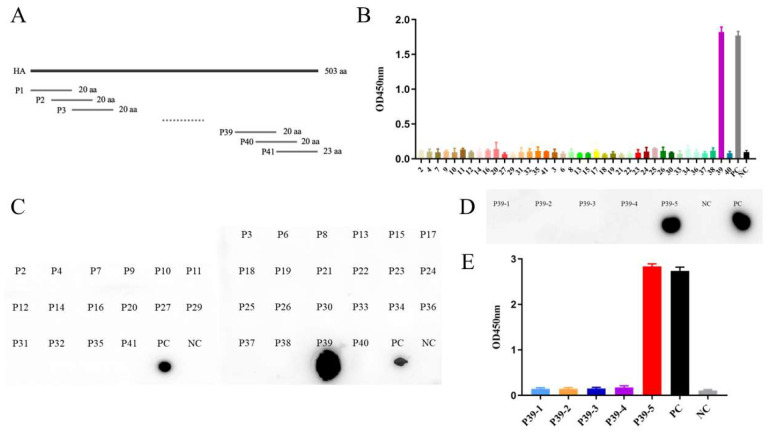
Identification of the B cell linear epitope on HA protein. (**A**) A schematic diagram of 41 overlapping peptides containing 20–23 amino acids (containing an offset of 7 aa) of HA protein (residues 19–521). (**B**) Reactivity of 41 overlapping peptides with mAb 6H2 detected by ELISA. (**C**) Dot blotting analysis of 41 overlapping peptides reacted with mAb 6H2. (**D**) Dot blotting results of five truncated peptides reacted with mAb 6H2. (**E**) ELISA results of five truncated peptides recognized by mAb 6H2. PC, recombinant HA protein diluted in peptide diluent. NC, peptide diluent.

To map the linear epitopes recognized by the two mAbs, 41 overlapping peptides containing an offset of seven amino acids were synthesized (Figure 4A and Table 1). The peptides binding to the mAbs were determined via dot blotting and indirect ELISA. As shown in Figure 4B,C, ^475^CFELYHKCDDQCMETIRNGT^494^ (P39) was recognized by mAb 6H2. To further confirm the precise epitope of the H9N2 HA protein, five truncated peptides of P39 were synthesized; the results of dot blotting and indirect ELISA showed that ^480^HKCDDQCM^487^ (P39-5) was recognized by mAb 6H2 (Figure 4D,E).

### 3.5. Biological Data Analysis

**Figure 5:**
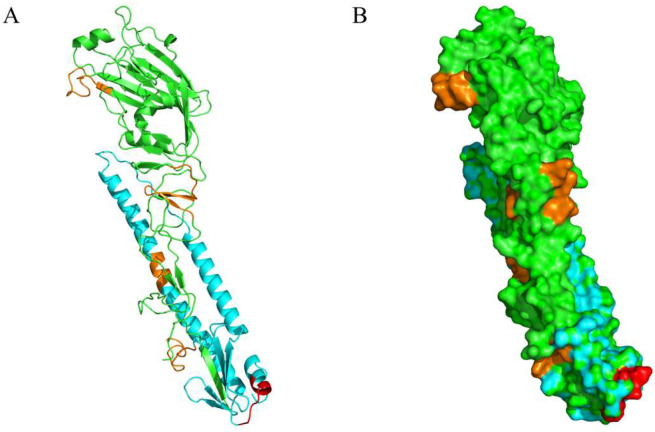
Spatial location of the identified epitope P39-5. (**A**) The location of the identified epitope using the hemagglutinin structure (PDB: 1JSD) as template by PyMOL. (**B**) Spatial distribution of the identified epitope in HA protein. ^480^HKCDDQCM^487^ (P39-5) is indicated in red, previously published epitopes are orange, HA1 is indicated in green, and HA2 is blue.

Structural analysis of the epitopes was performed using the PyMOL Molecular Graphics System; the hemagglutinin structure (PDB: 1JSD) was used as the template. The results showed that ^480^HKCDDQCM^487^ (marked in red) was located at the C-terminus of HA2, the bottom of HA, and near the disulfide bond-linked HA1 and HA2. It was distributed on the surface of the HA protein (Figure 5A,B).

### 3.6. Conservation Analysis of the Novel Epitope

**Figure 6:**
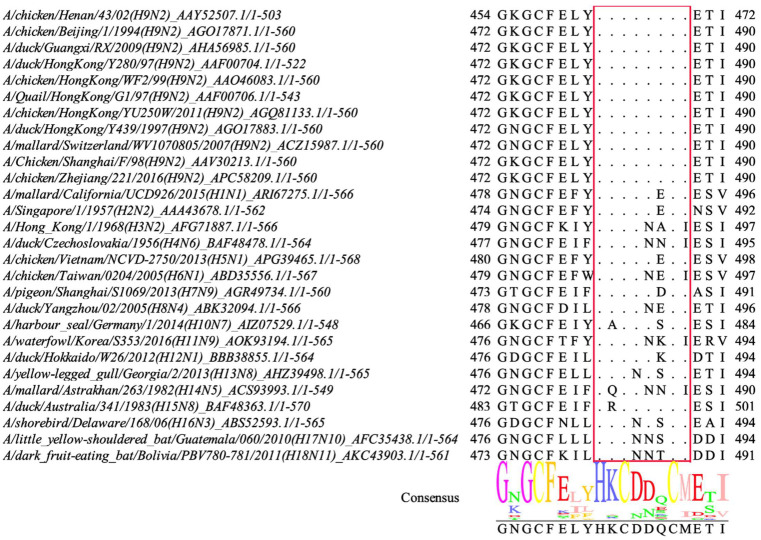
The conservation analysis of the identified epitope. Multiple sequence alignment of the identified epitope in 11 different strains of H9N2 AIV and H1-H18 AIV subtypes. The amino acid sequences of the novel identified epitope in different strains were highlighted with a red line.

To determine whether the identified novel epitope was conserved, 11 different H9N2 strains, primarily circulating strains derived from poultry and different AIV subtypes, were collected. The sequences of the HA proteins were then aligned using MEGA-X software and Jalview. As shown in Figure 6, the epitope ^480^HKCDDQCM^487^ was highly conserved among the various H9N2 strains. However, at least one amino acid showed differences among the other subtypes of the selected strains, indicating that the epitope was not conserved among other AIV subtypes.

## 4. Discussion

The AIV H9N2 has circulated widely in domestic poultry and spread worldwide since its first isolation from turkeys in 1966 [30,31,32,33]. H9N2 AIVs have been overlooked compared with the H5 and H7 subtypes; however, recent evidence has suggested that H9N2 AIV could play a central role in the next influenza pandemic or serve as an internal genetic donor to a pandemic virus [34]. H9N2 has become the dominant AIV subtype in chickens and ducks in China, replacing the AIVs H5N6 and H7N9 [8]. Vaccines remain the most effective approach to control AIV and enhancing the vaccination responses of vaccines is urgent [35,36]. However, mismatching vaccines and the emergence of antiviral drug resistance due to rapid antigenic variation limit the control of influenza.

Monoclonal antibodies are widely used in studies of H9N2 AIV for vaccines, therapeutics, diagnostic methods, and the identification of epitopes or key residues [13,21,37,38,39]. As the primary protective antigenic protein, HA is the major target of neutralizing antibodies [40]. In the present study, mice were immunized with rice-derived HA protein and positive hybridoma cells were successfully generated. The eight mAbs generated were screened using IPMA and ELISA, which suggested strong and specific reactions against both the H9N2 virus and HA protein, with the highest ELISA titer reaching 1:204,800. Among these mAbs, strains 3A5, 4E8, 8G8, and 12E5 showed robust hemagglutination-inhibitory activity, while results from the virus neutralization assay confirmed the strong neutralizing activity of 3A5, 4E8, and 8G8 via effective blocking of H9N2 AIV infection in MDCK cells, implying that the HA protein expressed by the rice endosperm system has the appropriate antigenic structure. Western blotting confirmed that two mAbs, 6H2 and 11B9, bound to the HA protein, indicating that these mAbs recognized linear epitopes of the H9N2 HA protein. To our knowledge, this is the first report of mAbs generated via immunizing mice with HA protein expressed in rice endosperm. Possibly due to the capacity of plants mediate complex post-translational modifications, such as glycosylation [41], some screened mAbs presented strong neutralizing and hemagglutination inhibition activities, and identifying new epitopes using the mAbs is promising.

Next, the linear epitopes recognized by the mAb 6H2 were mapped using 41 overlapping peptides derived from the HA protein sequence (residues 19–521) of the A/chicken/Henan/43/02 (H9N2) strain. These epitopes were evaluated via dot blotting and indirect ELISA, which showed that ^475^CFELYHKCDDQCMETIRNGT^494^ (P39) was recognized by the mAb 6H2. To further confirm the precise epitope, five truncated peptides were synthesized based on previous results and requirements for synthesis, and the epitope ^480^HKCDDQCM^487^ (P39-5) was recognized. According to the published B cell linear epitopes of the H9N2 HA, epitope ^284^GNCVVQCQTERGGLN^298^, located in HA1, presented weak immunogenicity. ^225^IGPRPLVNGLQGRI^238^ was located at HA1 and conserved among several influenza A viruses [42,43]. The epitope ^330^GIFGAIAGFIEGGW^344^, located in the fusion peptide, was predicted via a bioinformatic approach and was reported to be conserved among all influenza A subtypes [44]. ^433^NAELLVL^439^ was identified using mAb 3C12 with broad-spectrum activity and was generated through immunizing mice with the HA2 protein; the epitope is located at Helix C of HA2 and conserved among group 1 influenza A virus [45]. Hence, to our knowledge, the epitope ^480^HKCDDQCM^487^ (P39-5) is a novel B cell linear epitope of H9N2 HA.

Subsequently, the conservation of the identified epitopes was analyzed. To date, H9N2 viruses isolated from poultry in China have mainly been divided into the following sub-lineages: G1-like, Y439-like, Y280-like, BJ94-like, and F98-like [46]. HA sequences, with 11 different H9N2 strains of the above sub-lineages and different AIV subtypes H1–18, were aligned using MEGA-X software and Jalview. The results showed that the novel epitope, ^480^HKCDDQCM^487^, was highly conserved among the H9N2 strains. Thus, it is the first conserved B cell linear epitope of H9N2 subtype AIV HA and provides an ideal candidate for the differential diagnosis of H9N2 AIV.

Moreover, spatial analysis illustrated that the novel epitope ^480^HKCDDQCM^487^ was located at the C-terminus of the HA2 stem region. Previous reports have concluded that most broad-spectrum neutralizing antibody epitopes of the influenza virus are located at the periphery of the HA stem, preventing conformational changes by binding with antibodies and impeding the viral membrane fusion process, thereby playing an antiviral role [47,48]. As a representative membrane fusion protein, influenza virus HA is a homotrimeric class I transmembrane protein, similar to human immunodeficiency virus-1 (HIV-1) and Ebola Class I fusion proteins, with central coiled-coil motifs as the basic essential feature for fusion [49]. Comparing its crystal structures from the pre-fusion to the post-fusion states, HA2 undergoes tremendous changes; however, the intermediate structures between the two states remain poorly understood. In a widely proposed pathway, the globule domain of HA2, which comprises the identified novel linear epitope, sufficiently unfolds, subsequently zippers up along the formed coiled-coil, and plays an important role in membrane fusion [50]. In addition, fusion-inhibitory peptides targeting HA and HIV gp41 bind to the extended intermediate of the fusion protein, which supports this proposed pathway [51,52]. The exact role and structure of the C-terminal globule domain of the HA2 stem region remain unknown. The novel B cell linear epitope ^480^HKCDDQCM^487^ is the first epitope discovered to be located at the C-terminus of the HA2 stem region and warrants further studies.

Taken together, a panel of eight HA-specific mAbs was produced via immunizing mice with the HA protein expressed in rice endosperm, of which three showed strong neutralizing activity. Here, we report, for the first time, ^480^HKCDDQCM^487^, a novel B cell linear epitope located at the C-terminus of HA2, and is conserved among multiple H9N2 AIV strains. These findings provide insights into designing differential diagnostics for influenza viruses and vaccine development.

## Figures and Tables

**Table 1:** Amino acid sequence of peptide P1–P41.

Name	Peptide	Length (aa)
P1	CDKICIGYQSTNSTETVDTL	20
P2	CTETVDTLTENNVPVTHAKE	20
P3	CPVTHAKELLHTEHNGMLCA	20
P4	CHNGMLCATNLGHPLILDTC	20
P5	CPLILDTCTIEGLIYGNPSC	20
P6	CIYGNPSCDLLLGGREWSYI	20
P7	CGREWSYIVERPSAVNGLCY	20
P8	CAVNGLCYPGNVENLEELRS	20
P9	CNLEELRSLFSSARSYQRIQ	20
P10	CRSYQRIQIFPDTIWNVSYS	20
P11	CIWNVSYSGTSRACSDSFYR	20
P12	CSDSFYRSMRWLTQKNNAYP	20
P13	CQKNNAYPVQDAQYTNNRGK	20
P14	CYTNNRGKNILFMWGINHPP	20
P15	CWGINHPPTDTAQTNLYTRT	20
P16	CTNLYTRTDTTTSVATEDIN	20
P17	CVATEDINRTFKPLIGPRPL	20
P18	CLIGPRPLVNGLQGRIDYYW	20
P19	CGRIDYYWSVLKPGQTLRVK	20
P20	CGQTLRVKSNGNLIAPWYGH	20
P21	CIAPWYGHILSGESHGRILK	20
P22	CSHGRILKTDLNSGNCVVQC	20
P23	CGNCVVQCQTERGGLNTTLP	20
P24	CGLNTTLPFHNVSKYAFGNC	20
P25	CKYAFGNCPKYVGVKSLKLA	20
P26	CVKSLKLAVGLRNVPARSSR	20
P27	CVPARSSRGLFGAIAGFIEG	20
P28	CIAGFIEGGWSGLVAGWYGF	20
P29	CVAGWYGFQHSNDQGVGMAA	20
P30	CQGVGMAADRDSTQKAIDKI	20
P31	CQKAIDKITSKVNNIVDKMN	20
P32	CNIVDKMNKQYEIIDHEFSE	20
P33	CIDHEFSEVETRLNMINNKI	20
P34	CNMINNKIDDQIQDIWAYNA	20
P35	CDIWAYNAELLVLLENQKTL	20
P36	CLENQKTLDEHDANVNNLYN	20
P37	CNVNNLYNKVKRALGSNAVE	20
P38	CLGSNAVEDGKGCFELYHKC	20
P39	CFELYHKCDDQCMETIRNGT	20
P40	CETIRNGTYNRRKYKEESRL	20
P41	CYKEESRLERQKIEGVKLESEGT	23

**Table 2:** Amino acid sequence of truncated P39.

Name	Peptide	Length (aa)
P39-1	LGSNAVEDGKGC	12
P39-2	FELYHKC	7
P39-3	DDQCM	5
P39-4	CETIRNGT	8
P39-5	HKCDDQCM	8

**Table 3:** Characteristics of mAbs.

NO.	mAbs	Types	IPMA Titer	ELISA Titer	Ka (mol/L)
1	3A5	IgG1, Kappa	51,200	102,400	2.49 × 10^5^
2	4E8	IgG1, Kappa	12,800	51,200	4.17 × 10^4^
3	5A5	IgG2a, Kappa	6400	12,800	2.83 × 10^4^
4	6H2	IgG1, Kappa	12,800	102,400	3.49 × 10^4^
5	8G8	IgG1, Kappa	102,400	204,800	1.21 × 10^4^
6	11B9	IgG2b, Kappa	25,600	51,200	6.7 × 10^3^
7	11E3	IgG1, Kappa	12,800	51,200	7.8 × 10^3^
8	12E5	IgG1, Kappa	102,400	102,400	1.1 × 10^4^

Note: All the data are repeated three times and the average values are calculated.

## Data Availability

Not applicable.
